# Ocular manifestation of HIV/AIDS and correlation with CD4+ cells count among adult HIV/AIDS patients in Jimma town, Ethiopia: a cross sectional study

**DOI:** 10.1186/1471-2415-13-20

**Published:** 2013-05-27

**Authors:** Sisay Bekele, Yeshigeta Gelaw, Fasil Tessema

**Affiliations:** 1 Department of Ophthalmology, College of Public Health and Medical Sciences, Jimma University, Jimma, Ethiopia; 2 Department of Epidemiology, College of Public Health and Medical Sciences, Jimma University, Jimma, Ethiopia

**Keywords:** CD+4 T cells, HAART, HIV/AIDS, HIV retinopathy, Ocular manifestation

## Abstract

**Background:**

HIV/AIDS is one of twenty first century’s challenges to human being with protean manifestation affecting nearly all organs of our body. It is causing high morbidity and mortality especially in sub-Saharan Africa with numerous ocular complications and blindness. The purpose of this study was to determine the patterns of ocular manifestations of HIV/AIDS and their correlation with CD4+Tcells count.

**Methods:**

A cross-sectional study was done on 348 HIV-positive patients presented to Anti-Retroviral Therapy clinics. Data were collected using face-to-face interview, clinical examination and laboratory investigation, and analyzed using SPSS version 13 software. Statistical association test was done and p<0.05 was considered significant. Other statistical tests like student *t*-test and logistic regression were also done.

**Results:**

Of 348 patients, 175 were on antiretroviral therapy and 173 were not on therapy. The mean duration of therapy was 27 months. The overall prevalence of ocular manifestations was 25.3%. The commonest ocular manifestation was keratoconjunctivitis sicca (11.3%) followed by blepharitis (3.2%), molluscum contagiosum (2.6%), conjunctival squamous cell carcinoma (2.3%), conjunctival microvasculopathy (2.3%), cranial nerve palsies (2%), herpes zoster ophthalmicus (HZO) (1.2%), and HIV retinopathy (0.6%). HIV retinopathy and conjunctival microvasculopathy were common in patient with CD4+ count of <200 cells/μl while HZO and molluscum contagiosum were common in patients with CD4+ count of 200–499 cells/μl. Prevalence of ocular manifestation was higher among patients on HAART (32.6%) than those patients not on HAART (17.9%) (p<0.05). There was statistically significant association between ocular manifestation and sex, CD4+Tcells count, and age (p<0.05). CD4+ count, <200 cells/μl and age >35 years were independent risk factors for ocular manifestations.

**Conclusion:**

The study showed that the prevalence of ocular manifestation of HIV/AIDS is lower than previous studies and could be due to antiretroviral therapy. Lower CD4 count is a risk as well as predictor for ocular manifestations.

## Background

Human Immunodeficiency Virus (HIV) and Acquired Immunodeficiency Syndrome (AIDS) have been a major public health problem since the first case report in 1980s, and two third of people living with HIV/AIDS live in Sub-Saharan Africa [1]. Even though the prevalence of HIV/AIDS in Ethiopia is declining [2,3], it is still the major public health problem with a prevalence of 2.3% [4]. The disease is having a diverse impact on human being: affecting the economy, social life, education and the health of people. Patients with HIV/AIDS suffer from wide varieties of complications that are related to the infection. No organ of the body is spared from the virus or related diseases. The eye is an organ with wide spectrum HIV-related manifestations. The ocular manifestations can be the presenting sign of a systemic infection in an otherwise asymptomatic HIV-positive person. The disease can have adnexal, anterior segment, posterior segment, orbital, and neuroophthalmic manifestations [5]. Blindness, due to HIV-related complications, is also one of the problems endangering the life of people living with HIV with prevalence ranging from 6.9%-23% [6,7]. The prevalence of HIV-related ocular manifestations increase as CD4+ T cells count decreases. Diseases like cytomegalovirus (CMV) retinitis, keratoconjunctival sicca, retinal and conjunctival microvasculopathy occur commonly when the CD4 cells count falls below 100 cells/mm^3^ and Kaposi’s sarcoma occurs when the CD4+T cells count falls below 500 cells/mm^3^[8]. Introduction of highly active anti-retroviral therapy (HAART) has changed the prevalence and pattern of HIV-related ocular manifestation [9]. Before the introduction of HAART, CMV retinitis was the commonest ocular manifestation affecting 30%-40% of HIV-infected individuals [5,10]. In the HAART era, it has been suggested that there has been an estimated 80% decrease in the incidence of CMV retinitis [10]. The incidence of Kaposi’s sarcoma has declined by an estimated 87% [11]. Retinal microvasculopathy and opportunistic retinal infections were also found to be lower [9,12]. In the HAART era clinical entities like immune recovery uveitis have appeared as a cause of concern related to blindness [10,13].

Only few studies were done in Ethiopia and the majority of these studies were in the pre-HAART era (before 2002) and the study population was symptomatic patients with advanced stage of the disease and hence doesn’t reflect the true picture of HIV-related ocular manifestation among HIV-positive in the presence of anti-retroviral therapy. The purpose of this study is, therefore, to describe the prevalence and pattern of ocular manifestation among HIV/AIDS patients and determine correlation with the CD4+T cell count.

## Methods

A cross-sectional study was conducted at two Anti-Retroviral Therapy (ART) clinics (Jimma University Specialized Hospital and Jimma health center) from October to November 2009. From a total of 5692 adult HIV/AIDS patients, a sample of 369 was taken using 95% CI using simple random sampling technique. Only adults were included in the study because of consent issues. Proportionate numbers of patients were selected from the two ART centers and 185 patients were taken from those on Highly Active Anti-Retroviral Therapy (HAART) and 184 patients from those not on HAART. Patients with additional medical problems like Diabetes mellitus, hypertension and ocular trauma, which can have overlapping manifestation with HIV/AIDS, were excluded from the study.

Data were collected using interview, clinical examination and laboratory investigation. Questionnaire and recording format were used for the interview and recording clinical examination findings. Before the actual data were collected, a pretest was done on 37 (10%) study subjects. The interview was conducted by trained ophthalmic nurses. Eye examinations included; best corrected visual acuity test, intraocular pressure measurement, adnexal examination, pupil, motility, alignment, anterior segment, dilated fundus examination, and cranial nerve function tests. All ophthalmic examinations were done by investigators. The following tools were used for ophthalmic examinations; Snellen visual acuity charts, Schiotz tonometer, indirect ophthalmoscope, slit lamp biomicroscope, 20D Volk lens, 90D Volk lens, and Goldmann three-mirror lens. To prevent cross contamination, the instruments were irrigated with tap water and then disinfected by soaking in absolute alcohol for 10 minutes and rinsed with tap water and allowed to dry for 10 minutes after each procedure. For dilated fundus examination 1% tropicamide with or without phenylephrine was used and to anaesthetize the surface of the eye during intraocular pressure measurement and gonioscopy, 1% tetracaine eye drop was used. For some patients, based on their clinical finding, toxoplasmosis and syphilis serology tests were done. Histopathologic technique was used to confirm cases of tumor. For all patients who did not have CD4+ T cell count within three months before data collection, CD4 count was done during the study and 238 had new CD4+ T cells count.

Data were analyzed using SPSS version 13 software. *X*^2^-test was used to see association and p<0.05 was considered significant. Other statistical tests like student *t*-test and logistic regression were also used to see associations and differences between variables.

The study was conducted following Helsinki declaration and after it was approved by the Ethical Committee of Jimma University. Informed consent was taken and only those who consented were studied.

## Results

Out of 369 sampled study subjects, 348 patients were studied with the response rate of 94%. The mean age of the study subjects was 31.9 years (SD± 8.96) and 62% (216) of patients were in the age group of 20–34 years. The majority of the patients (74.1%) seen were female. One hundred seventy five patients were on HAART and the rest (173) were not on HAART. The mean duration of HAART was 27 months (SD ± 16.2, range 1–74).

Of the 348 patients, 88 (25.3%) had ocular manifestation. The prevalence of ocular manifestation in males (33.3%) was higher than the prevalence in females (23.3%) and this difference was statistically significant (p<0.05) (Table 1). Ocular manifestation was common in the age group of 30–34 years (20.7%) followed by 25–29 years (19.5%), and 35–39 years (18.4%). However, this difference in distribution of ocular manifestation in different age groups was not statistically significant (p>0.05) (Table 1). The mean age of patients with ocular manifestation (33.92±9.5 years) was higher than the mean age of patients without ocular manifestation (31.2±8.7 years) and this difference was statistically significant (t=2.463, p<0.05).

**Table 1:** Distribution of demographic characteristics and ocular manifestation of study population, Jimma, Ethiopia

**Demographic characteristics**		**With ocular manifestation n (%)**	**Without ocular manifestation n (%)**	**P-value**
Sex	Male	30 (33.3)	60 (67.7)	0.041
	Female	58 (22.5)	200 (75.5)	
	Total	88 (25.3)	260 (74.7)	
Age	<20	2 (2.3)	8 (3.1)	0.23
	20-24	11 (12.5)	50 (19.2)	
	25-29	17 (19.3)	62 (23.8)	
	30-34	18 (20.5)	58 (22.3)	
	35-39	17 (19.3)	35 (13.5)	
	40-44	10 (11.4)	28 (10.8)	
	>45	13 (14.8)	19 (7.3)	
	Total	88 (25.3)	260 (74.7)	

The mean CD4+ T cells count for those patients on HAART and those who were not on HAART were 366.85 cells/ml (SD±236.58) and 367.59 (SD±218.25) respectively. This mean difference was not statistically significant (t=−0.030; p=0.117). Prevalence of ocular manifestation was higher among patients on HAART (32.6%) than among patients not on HAART (17.9%) (p<0.05). But there was no statistically significant difference existed for patients with lower CD4+ cells count whether they were on HAART or not (p=0.099).

Patients with ocular manifestation had less CD4+ T cells count compared to patients without ocular manifestation with mean CD4+ T cell count of 308.74 cells/μl, and 386.56 cells/μl respectively and this was a statistically significant difference (t=−2.736; p=0.007). Patients with CD4+T cell count of <200 cells/μl comprised 36.5% of patients with ocular manifestation and only 18.7% of patients without ocular manifestation were in this CD4+T cell count category (Table 2).

**Table 2:** Ocular manifestation and CD4+ T cell count among HIV/AIDS patients, Jimma, Ethiopia

**Ocular manifestation**	**CD4+ T cell count (cells/μl)**			**Total n (%)**	**p-value**
	**0-199 n (%)**	**200-499 n (%)**	**500+ n (%)**		
Yes	31 (36.5)	36 (42.4)	18 (21.2)	85 (25.3)	0.002
No	47 (18.7)	139 (55.4)	65 (25.9)	251 (74.7)	
Total	78 (23.2)	174 (52.1)	83 (24.7)	336 (100.0)	

Logistic regression was done with variables which had significant association with the presence of ocular manifestation. Male patients were 1.75 times more likely to have ocular manifestation compared to female patients and this was statistically significant (p<0.05; CI: 1.03-2.96). But when controlled for age and CD4+ T cell count, this was not statistically significant (OR (CI) =1.37(0.77-2.41), P>0.05). Patients of age >35 years old were 1.86 more likely to have ocular manifestation compared to patients of age ≤ 35 years old and it was statistically significant (p<0.027; CI: (1.07-2.30)). The odds of having ocular manifestation was 2.52 for patients with CD4+ T cells count less than 200 cells/μl compared to patients with a CD4+ T cell count of ≥200 cells/μl and it was statistically significant (p=001; CI: 1.45-4.38). Patients on ART were 1.86 times more likely to have ocular manifestation compared to those who were not on ART and it was statistically significant (p<0.05; CI: 1.10-3.13) (Table 3).

**Table 3:** Risk factors for ocular manifestation of HIV/AIDS among patients Jimma, Ethiopia

**Factors**		**Ocular manifestation**		**OR (unadjusted) (95% CI)**	**OR (adjusted) (95% CI)**	**P-value**
		**Yes, n (%)**	**No, n (%)**			
Sex^§^	Male	30 (33.3)	60 (65.9)	1.72 (1.02-2.92)	1.36 (0.77-2.39)	0.295
	Female	58 (22.5)	200 (77.5)	1		
Age^ɸ^	>35 years	35 (34.3)	67 (65.7)	1.90 (1.14-3.16)	1.86 (1.07-2.30)	0.027
	≤35 years	53 (21.5)	193 (78.5)	1		
CD4+T cell count^‡^	<200 cells/μl	31 (39.7)	47 (60.3)	2.49 (1.45-4.29)	2.52 (1.45-4.38)	0.001
	≥200 cells/μl	54 (20.9)	204 (79.1)	1		
HAART^ʈ^	Yes	57 (32.6)	118 (67.4)	2.21 (1.34-3.65)	1.86 (1.10-3.13)	0.020
	No	31 (17.9))	142 (82.1)	1		

§; adjusted for age and CD4+ cell count.

ɸ; adjusted for sex and CD4+ cell count.

ʈ; adjusted for age and sex.

‡; adjusted for age, sex, and CD4+ cells count.

Adnexal manifestation was seen in 44 (12.8%) patients. The commonest adnexal manifestation was blepharitis which was seen in 3.2% patients. Molluscum contagiosum, conjunctival squamous cell carcinoma and conjunctival microvasculopathy were seen in 2.6%, 2.3%, and 2.3% respectively (Table 4). Majority of patients with adnexal manifestation (48.8%) had CD4+ T cell count of < 200 cells/μl whereas 80.8% patients with no adnexal manifestation had CD4+ T cells count of >200 μl. Conjunctival microvasculopathy and conjunctival squamous cell carcinoma were common in patients with a CD4+ T cell count of <200 cells/μl. Molluscum contagiosum and herpes zoster ophthalmicus were common in patients with a CD4+ T cell count of 200–499 cells/μl (Table 4).

**Table 4:** Distribution of adnexal manifestation and CD4+ T cell count of the study subjects Jimma town, Ethiopia

**Adnexal manifestation**	**CD4+ T cell count**			**Total**
	**0-199**	**200-499**	**500+**	**n (%)**
	**n (%)**	**n (%)**	**n (%)**	
Blepharitis	2 (18.2)	4 (36.4)	5 (45.4)	11 (3.2)
Molluscum contagiosum	3 (33.3)	5 (55.6)	1 (11.1)	9 (2.6)
CV	7 (87.5)	1 (12.5)	0 (0)	8 (2.3)
Conjunctival SCC*	6 (75)	2 (25)	0 (0)	8 (2.3)
HZO	1 (33.3)	2 (66.7)	0 (0)	3 (0.9)
Kaposi of lid	1 (100)	0 (0)	0 (0)	1 (0.3)
Kaposi of conjunctiva	0 (0)	0 (0)	1 (100)	1 (0.3)
Others	1 (50)	0 (0)	1 (50)	2 (0.6)
Total	21 (48.8)	14 (32.6)	8 (18.6)	43 (12.8)

*SCC= squamous cell carcinoma.

^*^HZO= herpes zoster ophthalmicus.

^*^CV= conjunctival microvasculopathy.

Forty three (12.3%) patients had anterior segment manifestations; the commonest manifestation being Keratoconjunctivitis sicca which was seen in 11.5% of all patients. Only 2 (0.6%), and 1(0.3%) patients had infectious keratitis and uveitis respectively (Table 5). No statistically significant difference was seen in the distribution of anterior segment manifestation and CD4+ T cell count (p>0.05). Keratoconjunctivitis sicca was seen commonly in patients with a CD4+ T cell count of 200–499 cells/μl (42.6%) (Table 5).

**Table 5:** Anterior segment ocular manifestations with CD4+ T cell count, Jimma, Ethiopia

**Anterior segment manifestations**	**CD4+ T cell count**			**Total n (%)**
	**0-199**	**200-499**	**500+**	
	**n (%)**	**n (%)**	**n (%)**	
KCS^*^	9 (23.7)	20 (52.6)	9 (23.7)	38 (11.3)
Infectious Keratitis	0 (.0)	1 (50.0)	1 (50.0)	2 (0.6)
Uveitis	1 (100.0)	0 (0)	0 (0)	1 (0.3)
Total	10 (2.9)	21 (6.3)	10 (2.9)	4 1(12.2)

^*^ KCS= Keratoconjunctivitis sicca.

Posterior segment and neuro-ophthalmic manifestations were not common. Only 16 (5%) patients had either posterior segment or neuro-ophthalmic manifestations. Facial nerve palsy was seen in 6 (1.8%) patients and it was common in patients with a CD4+ T cell count of ≥200 cells/μl (Table 6). All patients with HIV retinopathy had CD4+ T cell count of <200 cells/μl. Toxoplasmosis retinochoroiditis was seen in patients with a CD4+ T cell count of 200–499 cells/μl.

**Table 6:** Neuro-ophthalmic and posterior segment manifestation with CD4+ T cell count Jimma, Ethiopia

**Neuro-ophthalmic and posterior segment manifestations**	**CD4+ T cell count**			**Total n(%)**
	**0-199 n(%)**	**200-499 n(%)**	**>500 n(%)**	
Facial nerve palsy	1 (20)	2 (40)	2 (40)	5 (1.5)
Optic neuritis or atrophy	1 (25)	2 (50)	1 (25)	4 (1.2)
Chorioretinal pigmented scar	1 (33.3)	2 (66.7)	0 (0)	3 (0.9)
Toxoplasmosis retinochoroiditis	0 (0)	1 (100)	0 (0)	1 (0.3)
HIV retinopathy	2 (100)	0 (0)	0 (0)	2 (0.6)
Cytomegal virus retinitis	0 (0)	0 (0)	0 (0)	0 (0)
Total	5 (1.5)	7 (2.0)	3 (0.9)	15 (4.5)

The majority of patients (97.23%) had visual acuity of >6/18 in either or both eyes. No patient was found to be bilaterally blind but 9 patients (2.6%) had monocular blindness (Table 7). Half of the monocular blindness was due to refractive error and only two patients had monocular blindness secondary to central retinal vein occlusion and toxoplasmosis retinochoroiditis.

**Table 7:** Distribution of visual acuity of study population, Jimma, Ethiopia

**Visual acuity**	**Right eye n (%)**	**Left eye n (%)**	**Both eyes n (%)**
<3/60	1 (0.3)	8 (2.3)	0 (0)
3/60-6/60	8 (2.3)	2. (0.6)	2 (0.6)
6/60-6/18	6 (1.7)	7 (2)	4 (1.2)
>6/18	333 (95.7)	331( 95.1)	331 (97.2)
Total	348 (100)	348 (100)	337 (100)

## Discussion

In this study, there was no record of WHO stage of the HIV/AIDS the study subjects and hence this information was not included in the analysis though we compared our findings with other studies. However, this will not have significant influence on the interpretation as the CD4+ T cell count is used as part of the parameter for staging and found to be correlated with WHO stage of HIV/AIDS [14].

The prevalence of ocular manifestation in this study was found to be 25.3% which is much lower than the study conducted in Gondar University Hospital [15] which was 60% but comparable to other studies in Africa (19% and 20%) [16,17]. This difference could be due to the nature of the study at Gondar which was conducted on patients who were admitted to hospital with a medical problem and came to the eye clinic with ocular complaint and 90% of patients were in WHO stage of III and IV. The fact that the Gondar study was conducted during the pre-HAART era might have contributed to the difference as it is known that HAART decreases the prevalence of HIV/AIDS related ocular diseases.

In this study, it was found that the mean CD4+ T cells count of patients with ocular manifestation (308.74 cells/μl) was lower than the mean CD4+ T cell count of patients without ocular manifestation (386.56 cells/μl). It was also found that ocular manifestation was common in patients with a CD4+ T cell count of <200 cells/μl. These findings are similar to other studies conducted in India [7] and Senegal [18]. There was, however, one peculiar finding in our study. Patients who were on HAART had a high prevalence of ocular manifestation compared to those who were not on HAART. This could be due to the low CD4+ T cells count during the initiation of HAART as patients with a CD4+ T cell count of <250 cells/μl are put on HAART. This low CD4 count is associated with higher prevalence of ocular manifestation as shown in this study and patients might have already developed the manifestation before initiation of HAART. Even though patients are on HAART and their CD+T cells count increases, the newly formed population of lymphocytes are not associated with functional maturity of the immune system and patients are not protected [8,19]. Our patients were on HAART for an average of 27 months and this is not enough for the functional maturity of the immune system as shown by Pakker *et ai*[20] that at around two years only a small proportion of individuals demonstrate immune reconstitution close to the normal range.

In this study it was found that age >35 years, and CD4+ T cell count of <200 cells/μl were independent risk factors for patients to develop ocular manifestations. CD4+ T cell count <200 cells/μl being a risk factor is a well-established fact [7,18] and the difference between the different age group could be due to the effects of age on the immunity of the patients.

The commonest ocular manifestations in this study were adnexal (12.8%) of which blepharitis was the commonest (3.2%) followed by molluscum contagiosum (2.6%), conjunctival Squamous cell carcinoma (2.3%) and conjunctival microvasculopathy (2.3%). This finding is different from the study conducted in Gondar University hospital [15] where the commonest ocular manifestations were HIV retinopathy (24%) followed by neuro-ophthalmic disorders (9.6%). This could be due to the fact that the study was done on patients with advanced stage of the disease (90% in stage III and IV) where HIV retinopathy and neuro-ophthalmic disorders are common. The distribution of adnexal manifestation with CD4+ T cell count is nearly similar to earlier studies [5,18] whereby Molluscum contagiosum and herpes zoster ophthalmicus occurred in CD4+ T cell count range of 200–499 cells/μl and conjunctival microvasculopathy occurred in patients with a CD4+ T cell count of <200 cells/μl.

The commonest anterior segment manifestation was Keratoconjunctivitis sicca (11.5%). The prevalence is similar to another study (10%-20%) [21].

Neuro-ophthalmic manifestations were seen in 2.6% of all patients which is lower than the finding of the Gondar study (9.6%) [15]. HIV retinopathy was seen in only 2 (0.6%) patients which is very much lower than previous studies (24%-40%) [15,22,23]. Other studies in Gambia (3%) [23] and India (5%) [6] showed reasonably lower prevalence of HIV retinopathy compared to the above studies, but still higher than this study. The studies with higher prevalence of HIV retinopathy were done on patients in the Pre-HAART era and majority of the study subjects were in advanced stages of the disease. HIV retinopathy was seen solely in patients with a CD4+ T cell count of <200 cells/μl and toxoplasmosis retinochoroiditis was seen in patients with a CD4+ T cell count of 200–499 cells/μl and this is similar to other studies [5,18]. There was no single case of CMV retinitis in this study. It was also found by other studies that CMV retinitis is rare among African patients [12,24,25]. This could be the reason for the low prevalence of immune recovery uveitis (0.3%) despite 50% of our study patients being on HAART.

## Conclusion

The overall prevalence of ocular manifestation of HIV/AIDS was lower than what is reported in previous studies but it is still reasonably high. The prevalence was higher among male patients, patients with lower CD4+ T cell count and older patients. The commonest ocular manifestations were anterior segment and adnexal manifestations. Posterior segment manifestations were rare and there was no case of CMV retinitis. Age >35 years, and CD4+ T cell count of <200 cells/μl were found to be independent risk factors for developing ocular manifestations.

Though ocular manifestations of HIV/AIDS are low, HIV/AIDS patients especially those with lower CD4+ T cell count and older patients should have eye checkup and follow up by an ophthalmologist; and there should be concerted care with a multidisciplinary approach. Further prospective study should be carried out to investigate why some ocular findings are rare in our setting so that the real clinical picture and possible reasons will be known. The association between age and ocular manifestation warrants further investigation.

## Abbreviations

ART: Antiretroviral therapy; AIDS: Acquired immunodeficiency syndrome; CD: Cluster of differentiation; CMV: Cytomegalovirus; HAART: Highly active antiretroviral therapy; HIV: Human immunodeficiency virus; HZO: Herpes zoster ophthalmicus.

## Competing interests

None of the authors have any proprietary interests or conflicts of interest related to this manuscript.

## Authors’ contribution

SB conceived, designed, acquired, analyzed and interpreted the data; drafted the manuscript and approved for publication. YG participated in the conception, design and interpretation of data; revised the manuscript critically and approved for publication. FT participated in the design and interpretation of data; revised the manuscript. All authors read and approved the final manuscript.

## Pre-publication history

The pre-publication history for this paper can be accessed here:

http://www.biomedcentral.com/1471-2415/13/20/prepub
